# LIM kinase1 modulates function of membrane type matrix metalloproteinase 1: implication in invasion of prostate cancer cells

**DOI:** 10.1186/1476-4598-10-6

**Published:** 2011-01-10

**Authors:** Tenekua Tapia, Richard Ottman, Ratna Chakrabarti

**Affiliations:** 1 Department of Molecular Biology and Microbiology, Burnett School of Biomolecular Sciences, University of Central Florida, Orlando, FL, USA

## Abstract

**Background:**

LIM kinase 1 (LIMK1) is an actin and microtubule cytoskeleton modulatory protein that is overexpressed in a number of cancerous tissues and cells and also promotes invasion and metastasis of prostate and breast cancer cells. Membrane type matrix metalloproteinase 1 (MT1-MMP) is a critical modulator of extracellular matrix (ECM) turnover through pericellular proteolysis and thus plays crucial roles in neoplastic cell invasion and metastasis. MT1-MMP and its substrates pro-MMP-2 and pro-MMP-9 are often overexpressed in a variety of cancers including prostate cancer and the expression levels correlate with the grade of malignancy in prostate cancer cells. The purpose of this study is to determine any functional relation between LIMK1 and MT1-MMP and its implication in cell invasion.

**Results:**

Our results showed that treatment with the hydroxamate inhibitor of MT1-MMP, MMP-2 and MMP-9 ilomastat inhibited LIMK1-induced invasion of benign prostate epithelial cells. Over expression of LIMK1 resulted in increased collagenolytic activity of MMP-2, and secretion of pro-MMP2 and pro-MMP-9. Cells over expressing LIMK1 also exhibited increased expression of MT1-MMP, transcriptional activation and its localization to the plasma membrane. LIMK1 physically associates with MT1-MMP and is colocalized with it to the Golgi vesicles. We also noted increased expression of both MT1-MMP and LIMK1 in prostate tumor tissues.

**Conclusion:**

Our results provide new information on regulation of MT1-MMP function by LIMK1 and showed for the first time, involvement of MMPs in LIMK1 induced cell invasion.

## Introduction

LIM kinase 1 (LIMK1) is a downstream effector of Rho signaling pathway, which modulates actin dynamics. LIMK1, a unique serine/threonine kinase containing two N-terminal LIM domains in tandem and a PDZ domain [1] is a newly identified candidate that promotes prostate and breast cancer metastasis [2-4]. High levels of LIMK1 have been observed in highly invasive prostate cancer cell lines and in human prostate tumors [2,3,5]. LIMK1 expression increased invasiveness of non-invasive prostate and breast cancer cells and expression of antisense RNA or dominant negative kinase-dead LIMK1 greatly reduced invasion of prostate and breast cancer cells [2-4]. LIMK1 regulates actin cytoskeleton remodeling through inactivating phosphorylation of cofilin on Ser^3 ^residue [6] resulting in accumulation of actin polymer. The catalytic activity of LIMK1 requires activating phosphorylation at the T^508 ^residue in its kinase domain, which changes conformation of the kinase domain and favors dissociation of the autoinhibitory N-terminal LIM domains from the C-terminal kinase domain making the kinase domain accessible to its substrate [7]. Activating phosphorylation of LIMK1 is mediated by p21 kinase (PAK1 & PAK4) and Rho kinase (ROCK), which in turn are activated by the members of Rho subfamily of small GTPases (Rho, Rac and Cdc42) [8]. LIMK1 is also involved in Rac-mediated lamellipodia formation [9].

Membrane type matrix metalloproteinase 1 (MT1-MMP) belongs to a family of zinc binding collagenase that is involved in extracellular matrix (ECM) turnover [10]. The ability of MT1-MMP to degrade ECM has established its role in physiological and pathological tissue remodeling such as angiogenesis and tumor development. Expression of MT1-MMP is documented in various tumor cells and strongly implicated in tumor progression and metastasis [11]. MT1-MMP shares conserved structural features with other MMPs, such as an N-terminal signal peptide, a propeptide and a catalytic domain [12]. In its active form MT1-MMP is a membrane-tethered metalloproteinase, which anchors to the plasma membrane with its transmembrane domain so that the catalytic domain is exposed on the surface of the cells [13].

Activation of MT1-MMP requires removal of the propeptide by furin convertase, resulting in a 57 kDa active enzyme [14] and its targeting into the plasma membrane. Tissue inhibitor of matrix metalloproteinase 2 (TIMP-2) interacts with the membrane-tethered MT1-MMP with its catalytic domain and inhibits its proteolytic activity [15]. MT1-MMP bound with TIMP-2 acts as a receptor for binding of soluble pro-MMP-2 with its hemopexin domain. The trimolecular complex of MT1-MMP/TIMP-2/pro-MMP-2 then present pro-MMP-2 to a neighboring TIMP-2 free MT1-MMP, which cleaves pro-MMP2 to its active form [16]. To position another molecule of MT1-MMP next to the ternary complex, MT1-MMP forms a homo-oligomeric complex through its hemopexin and or transmembrane/cytoplasmic domain [17,18]. Recent studies linked the function of MT1-MMP and MMP-2 on ECM degradation and metastasis by showing the processing [19], membrane targeting [20], autocatalysis [21] and internalization [22] of MMPs. These studies showed that MT1-MMP and MMP-2 function through balanced activation and inactivation process and any alteration in the activation and processing of MMPs influence the overall maintenance of ECM homeostasis, which may trigger excessive ECM degradation leading to cancer metastasis. MT1-MMP/TIMP-2/MMP-2 activation complex also processes proMMP-9 to its active form, which is mediated by TIMP-2-regulated cascade of zymogen activation initiated by MT1-MMP [23]. Recent studies also showed activation of MMP-9 by an MT1-MMP associated protein through RhoA activation and actin remodeling [24]. Because MT1-MMP, MMP-2 and MMP-9 are all overexpressed in invasive prostate cancers, it is likely that increased activation of MT1-MMP/MMP-2 complex also activates proMMP-9 and acts as a major mediator of pericellular proteolysis [13,25].

Earlier studies showed the involvement of activated Rac1 and RhoA in induction of metastasis in animals suggesting that the signaling pathway regulated by these proteins may play a role in acquisition of the metastatic phenotype [26]. Rac1 is essential for growth factor-induced cell invasion and lamellipodia formation through modulation of actin cytoskeleton [27]. Later on, the role of Rac1 in tumor cell invasion mediated through expression, processing and activation of MMPs was established [28]. These observations indicate a possible link between activation of MMP and LIMK1 function. In this study, we examined the involvement of MMPs in cell invasion induced by LIMK1 and the role of LIMK1 in regulation of expression and activation of MT1-MMP in prostate epithelial cells.

## Materials and methods

### Cell lines and antibodies

The parental BPH-1 cells (a gift from P Narayana, University of Florida) [29], and its transfected sub-lines, BPHL^CA ^and BPH^V^, were maintained in Dulbecco's modified Eagle's medium (Sigma-Aldrich). PC3 cells (ATCC) were maintained in HAM 12 medium (Sigma-Aldrich). All media were supplemented with 10% fetal bovine serum, 2 mM glutamine and 1× antibiotic and antimycotic solution (Invitrogen). BPHL^CA ^and BPH^V ^cells were developed by stable transfection of constitutively active (phosphomimic mutant) LIMK1 gene containing a Flag tag at the 3' end cloned in pRevTRE (Clontech, Mountain View, CA), and an empty vector, respectively. A number of hygromycin resistant clones were isolated and mixed for subsequent experiments to avoid clonal bias. The phosphomimic mutant of human LIMK1 (LIMK^T508EE^) was generated by site directed mutagenesis of T^508 ^to EE. Transfected cells were routinely maintained in antibiotic (hygromycin) containing media. All cells were grown in appropriate growth media in a humidified atmosphere containing 5% CO_2_, at 37°C. Monoclonal or polyclonal antibodies specific for LIMK1 (BD Biosciences and Santa Cruz Biotechnology), Trans-Golgi Network Golgi marker TGN46 (Novous Biologicals, Littleton CO). MT1-MMP (Neomarker, Fremont, CA, Thermo Fisher, Rockford, IL, and Chemicon, Millipore, Billerica, MA) and Flag (Sigma) were used for various experiments.

### Tumor samples

Human multiple prostate cancer tissue microarrays (TMA) (PR483) from US Biomax, Inc. (Rockville, MD) were used to detect expressions of MT1-MMP and LIMK1. PR483 contained 40 cases of prostate cancer tissues with 8 cases of normal tissues from autopsy. TMA containing formalin fixed and paraffin embedded tissue samples were cut at 5 μm thickness and mounted on positively charged SuperFrost Plus glass slides. Individual cores in TMA sections were 1.5 mm in diameter. All tumors were malignant in nature with Gleason Scores ranged between 2(1+1) to 10(5+5) and with no detectable local invasion or metastasis. TNM classification showed that tumors in the TMA were either T2NxM0 or T3NxM0 or T4NxM0. The composition of tumor tissues included 20 tumors of grades between 1 and 2 (low grade LG) and 20 tumors of grades between 3-4 (high grade HG). Tumor tissues were obtained from patients with ages ranged from 20-87 years.

### Invasion assay

BPHL^CA ^and BPH^V ^cells were maintained in phenol red-free medium and were seeded at a density of 1.25 × 10^5 ^in serum-free medium in the upper chamber containing matrigel coated inserts (8 μM pore) of the *in vitro *invasion chamber (ECM 554, Chemicon). EGF was added to the cell suspension at a final concentration of 10 ng/ml. Serum free media containing EGF (100 nM) was added to the bottom chamber as the chemoattractant and chambers were incubated at 37°C in a CO_2 _incubator for 48 hrs. Parallel experiments were performed in the presence or absence of GM6001 (Ilomastat, Chemicon) at a concentration of 25 μM in serum free media. Next, cells migrated to the inner side of the inserts were detached, stained with fluorescent dye solution and lysed. Fluorescence was measured in a Wallac Victor 2 spectroflurimeter. Cells that traversed through the matrigel and accumulated in the bottom chamber were also counted. Invasion was confirmed by staining the underside of the membrane with 0.1% Crystal violet solution. Data was calculated as fold changes in the averaged values obtained from relative fluorescence unit (RFU) and cell enumeration.

### Gelatin Zymography

Transfected cells (2.5 × 10^5^) cells were seeded in equal volumes of culture media into six well dishes and incubated for 24 hrs. Next day, media was replaced with phenol red free DMEM supplemented with 10% charcoal stripped FBS and cells were incubated at 37°C in a CO_2 _incubator for 48 hrs. Cells were then serum starved for 24 hrs; conditioned media were collected and centrifuged at 500 × g for 5 mins at 4°C. The supernatants were separated and used for zymography. Equal volume of each sample with or without concentration was incubated with non-reducing loading buffer at room temperature for 15-20 mins. Samples were then separated on a 10% SDS gel co-polymerized with gelatin (1 mg/mL). Next, gels were incubated in renaturing buffer (2.5% Triton X-100 in distilled water) for 30 mins, washed and incubated in developing buffer (50 mM Tris pH 7.8, 0.2 M NaCl, 5 mM CaCl_2_, 0.02% NP-40) at 37°C for 24 hrs. Gelatinoic bands were visualized by staining with Coomassie blue followed by destaining of the gels and quantified by densitometric analysis of the dried gels using Gene Snap software.

#### Quantitative Real time PCR

Total RNA from BPHL^CA ^and BPH^V ^cells was extracted using a total RNA extraction kit (Promega, Madison, WI) and used for quantitative real time PCR. For cDNA synthesis, BioRad iScript kit was used according to manufacturer's protocol. Briefly, RNase free water and 5× reaction mix (provided with kit) were added to total RNA samples (1 μg). Next, samples were denatured at 65°C for 15 mins and cooled to 37°C for 3 mins. Reverse transcription was carried out at 42°C for 1.5 hrs. Real-time PCR was performed using a SYBR green based PCR kit (Biorad) and cDNAs. A RT-PCR reaction without reverse transcriptase was used as a control. Specific primers for MMP-2 (F: 5'GTCTCCTGCTCCCCCT3', R: 5' CGAACATTGGCCTTGATCTCA3') and GAPDH (F: 5'GCAAGTTTCCGTTCCGCTTCC3', R: 5'CAGTACCAGTGTCAGTATCAGC3') were used for QPCR (40 cycles) of MMP-2 and GAPDH transcripts. Reactions were carried out in BioRad iCycler thermocycler. Quantification of the relative expression of MMP-2 gene was performed using 2^-ΔΔCt ^method and GAPDH as a reference gene. To calculate relative expression, MMP-2 expression was normalized for each sample using GAPDH expression. Fold change expression was calculated as a ratio of normalized expression of MMP-2 in BPHL^CA ^cells and in BPH^V ^cells.

### Immunoblot and immunoprecipitation

Total cell extracts from BPH-1, BPHL^CA^, BPH^V ^and PC3 cells were prepared using the lysis buffer (50 mM Tris pH 8.0, 120 mM NaCl, 2.5 mM EDTA, 1 mM PMSF, 1%NP-40, 10 μg/mL leupeptin/aprotinin) and freeze-thaw cycles. Total proteins (50 μg) were separated in SDS-PAGE and subjected to immunoblot analysis using primary antibodies against the Flag tag, LIMK1 or MT1-MMP to monitor expression of specific proteins. A chemiluminescence detection kit (Thermo Scientific, Rockford, IL) was used to detect target proteins using corresponding secondary antibodies. For immunoprecipitation, crude PC3 cell extracts were diluted in RIPA buffer containing proteinase inhibitor mixture set III (Calbiochem EMD, Gibbstown, NJ) and treated with antibodies against LIMK1 or MT1-MMP using the standard protocol. Antigen-antibody complexes were immunoprecipitated using protein A/G PLUS sepharose beads (Santa Cruz Biotechnology) and detected by immunoblot analysis using specific antibodies.

### Gene silencing using small interfering RNA

Inhibition of LIMK1 expression in PC3 cells was conducted by transfection of HuSH shRNA constructs against LIMK1 (AAGGACAAGA  GGCTCAACTTCATCACTGA) in pGFP-V-RS vector (Origene Technologies). Initially four different shRNAs of LIMK1 were screened to identify the shRNA that caused maximum inhibition of LIMK1 expression for subsequent experiments. An shRNA construct for scrambled RNA was used to evaluate the off target effect of the shRNA. Cells were transiently transfected using Lipofectamine LTX (Invitrogen, Carlsbad, CA) reagent or FuGENE HD and shRNA constructs, and incubated for 55-72 hrs for optimum knockdown of LIMK1.

### Immunohistochemistry (IHC)

The TMA sections were deparaffinized in xylene, hydrated with a graded series of alcohol (100%, 95%, and 80% ethanol [vol/vol] in deionized H_2_O), and re-hydrated in de-ionized water. Sections were incubated for 5 mins in 3% H_2_O_2 _in water to block endogenous peroxidase and washed. Antigen retrieval was achieved by placing slides in 1× antigen retrieval solution (Target Retrieval solution, S-1699, DakoCytomation) for 30 mins in microwave oven with simmering conditions then cooled down for 15 mins at room temperature. Slides were then washed with PBS that contained 0.1% triton and 0.1% BSA. Nonspecific binding was blocked with (2.5%) normal horse blocking serum and 2% BSA in PBS. The slides were then incubated for 1 hr at room temperature with one of the following: 1) monoclonal mouse anti-LIMK1 (1:600 dilution) or 2) rabbit anti-MMP-14/MT1-MMP antibodies (Millipore Ab-1) (1:800 dilution). Slides were then washed with PBS that contained 0.1% triton and 0.1% BSA. Slides were then incubated with ImmPRESS™ Reagent anti-Rabbit or anti-Mouse Ig (peroxidase)(Vector Laboratories) for 30 mins at room temperature. Slides were washed next and incubated in peroxidase substrate DAB solution (DAKO Cytomation). Finally, sections were washed in tap water and counterstained with Hematoxylin QS (Vector Labs). Slides were mounted with permanent mounting medium (C0487, Sigma). IHC staining was evaluated by an independent pathologist from US Biomax, Inc. Manual scoring of intensity, negative (0), weak (1+), moderate (2+), or strong (3+), location and cell types of staining were performed by the pathologist and the scores were then converted to number from 0 to 3 scales. Images of the stained sections were scanned and the total positive cell numbers and intensity of anti-LIMK1 and anti-MT1-MMP staining were computed and measured by ImageScope from Aperio Scanning System (US Biomax, Inc).

#### Dual and triple label Immunofluorescence analysis

BPHL^CA ^and PC3 cells were plated on poly-L lysine coated glass coverslips in 24 well culture dishes in complete growth medium for 24 hrs. In some cases, PC3 cells were transfected with plasmids containing cDNA for LIMK1 shRNA or nonspecific shRNA and maintained for an additional 24 hs. For immunostaining, cells were washed in phosphate buffer (0.1 M) (PB) and fixed in 4% paraformaldehyde in PB for 10 mins at room temperature. Next, cells were washed with 0.2% TritonX-100 (BPH cells) or 0.1% Triton X-100 (3 mins) and 0.1% Tween 20 (4 mins) (PC3) in PB. Cells were next subjected to blocking at room temperature in 10% goat serum, 0.2% triton X100 in PB for 1.5 hrs. Primary antibodies in blocking buffer (Flag 35 mg/ml, MT1-MMP 1:200, LIMK1 1:50, and TGN46 1:115) were combined and applied to coverslips for 1 hr. Respective secondary antibodies conjugated with Alexa 647, Cy 3 or Cy 5 were used next for 30 mins at room temperature. Coverslips were washed with PB, postfixed (4% paraformaldehyde) for 5 mins and mounted with gel mount (BioMeda, Foster City, CA). Cells were visualized in a Zeiss 710 confocal microscope. For quantification of colocalization, specific regions of singly labeled cells were selected first to set the thresholds. Then selected regions of interest, either of individual vesicles, entire cell, or entire membrane were used for pixel quantification. Colocalization of proteins was quantified using Zeiss Zen 2009 software or Olympus FV1-ASW software, which calculates overlap and colocalization coefficient as derived from Mander's article based on Pearson's correlation coefficient.

$$\text{Overlap Coefficient}:\;\;\;\left[\Sigma \left(\text{Ch}1_{\text{i}}\right)\left(\text{Ch}2_{\text{i}}\right)\right]/\left[\surd \left(\text{sum}{\left(\text{Ch}1_{\text{i}}\right)}^{2}{\left(\text{Ch}2_{\text{i}}\right)}^{2}\right)\right]$$

The values for the overlap coefficient range from 0 to 1. An Overlap Coefficient with a value of 1 represents perfectly colocalized pixels.

$$\text{Pearson's Correlation}:\;\left[\Sigma \left(\text{Ch}1_{\text{i}}-\text{Ch}1_{\text{avg}}\right)\left(\text{Ch}2_{\text{i}}-\text{Ch}2_{\text{avg}}\right)\right]/\left[\surd \left(\text{sum}{\left(\text{Ch}1_{\text{i}}-\text{Ch}1_{\text{avg}}\right)}^{2}{\left(\text{Ch}2_{\text{i}}-\text{Ch}2_{\text{avg}}\right)}^{2}\right)\right]$$

Because each pixel is subtracted by the average pixel intensity, the value for Correlation R can range from -1 to 1. A value of 1 would mean that the patterns are perfectly similar (colocalized), while a value of -1 would mean that the patterns are perfectly opposite.

### Surface staining of MT1-MMP and cell surface biotinylation

BPHL^CA ^or BPH^V ^cells were seeded in complete growth medium to 80% confluence and harvested by incubating in cell stripper (Cell Gro, Manassas, VA) at room temperature. Phosphate buffered saline (PBS) was added to the dish and cells (5 × 10^5^) were collected by centrifugation. Cells were suspended in PBS containing 3%FBS and MT1-MMP antibodies against the extracellular catalytic domain (Chemicon) (5 μg/1 × 10^6 ^cells) and incubated at 4°C with rocking for 3 hrs. Cells were washed with PBS containing 3% FBS and incubated with secondary antibodies conjugated with Alexa 488 (Molecular Probes, Carlsbad, CA) (1:800) for 30 mins with rocking at 4°C. Cells were washed and fixed with sterile 2% paraformaldehyde in PBS. Cells were analyzed in a flow cytometer (FACS Calibur/BD Biosciences, San Jose, CA). For biotin labeling of cell surface proteins, PC3 cells (3 × 10^5 ^cells/well) were seeded on 6-well dishes and after 24 hrs transfected with cDNAs for LIMK1 shRNA or control shRNA. After 68 hrs of incubation, cells were incubated with cell impermeable Ez-Link Sulfo-NHS-LC-Biotin (Pierce) (0.5 mg/ml) at 4°C with rocking for 30 mins and quenched with 100 mM glycine to remove excess Biotin according to the method described in [30,31]. Next, cells were harvested in RIPA Buffer (50 mM Tris, pH 7.4, 150 mM NaCl, 5 mM EDTA, 1% Triton X-100 and 0.1% SDS) with proteinase inhibitors (1 μg/ml aprotinin, 1 μM pepstatin, and 10 μM leupeptin) by scraping. Cells were lysed and clarified by centrifugation. Biotin-labeled surface proteins were separated from equal amounts of cell lysate proteins, by incubating with washed UltraLink Streptavidin sepharose beads (Pierce) at 4°C with mixing for 14 hrs. Bead-bound proteins were separated on SDS-PAGE and immunoblotted for MT1-MMP (1:500) on the plasma membrane using antibodies against the hinge region (Millipore).

### Dual Luciferase reporter assay

The MT1-MMP promoter-luciferase construct containing firefly luciferase driven by a 7.2 KB promoter fragment of MT1-MMP (kindly provided by Jorma Keski-Oja, University of Helsinki) was used for transient transfection using Lipofectamine LTX according to our published protocol [32]. A construct containing Renilla Luciferase driven by thymidine kinase promoter was used for cotransfection as the transcription control. Transfected BPH-1 sublines and PC3 cells with or without co-transfection of cDNAs for LIMK1 shRNA or control shRNA were used for luciferase reporter assays. Cells were harvested at 62 hrs post transfection and luciferase expression was determined using a Dual luciferase assay kit (Promega) according to supplier's protocol.

### Statistical analysis

Quantitative results are presented as meam ± SD of the number of independent experiments performed. Statistical differences were calculated using Student's t-test in GraphPad/Prism 4.0a. A *p *value of < 0.05 was considered significant. IHC scoring data were analyzed using GraphPad/Prism 4.0a.

## Results

### LIMK1-induced invasion is mediated by MMPs

**Figure 1:**
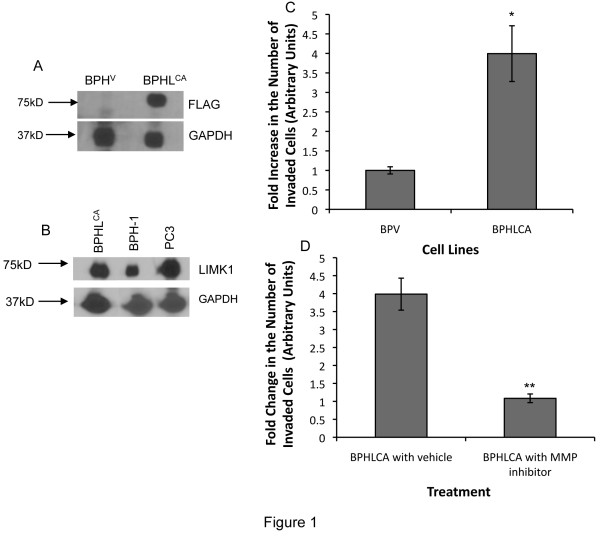
**MMP inhibitor suppressed invasiveness of BPH cells expressing LIMK1**. A) Immunoblot analysis of expressed Flag-tagged LIMK1 in transfected BPH-1 cells (BPHL^CA^) using anti-Flag antibodies. No cross reactivity was noted in vector transfected (BPH^V^) cells. B) Comparative analysis of LIMK1 expression in PC3, parental BPH-1 and BPHL^CA ^cells using anti-LIMK1 antibodies showing increased expression of LIMK1 but at a physiological level in BPHL^CA ^cells. C and D). In vitro invasion assays of BPHL^CA ^and BPH^V ^cells with or without treatment with MMP inhibitor GM6001. C) Fold increase in the number of invaded cells expressing LIMK1 (BPHL^CA^) compared to the vector control (BPH^V^). D) Fold changes in cells treated with GM6001 that invaded through the matrigel membrane compared to the vehicle treated BPHL^CA ^cells. Values are Mean ± SD of three separate experiments. *P < 0.003, **P < 0.002 (BPH^V ^vs. BPHL^CA ^cells).

MT1-MMP, MMP-2 and MMP-9 are often overexpressed in advanced prostate cancers and play essential roles in prostate tumor metastasis [33,34]. Because LIMK1 expression was sufficient to sponsor an invasive phenotype of BPH-1 cells [2] we sought to determine if LIMK1-induced invasion is mediated by these MMPs. We used transfected BPHL^CA ^cells expressing Flag-tagged constitutively active LIMK1 (Figure 1A). We chose BPH-1 cells, which was originally isolated from benign prostatic hyperplasia, for ectopic expression of LIMK1 as these cells express low levels of LIMK1 compared to the metastatic prostate cancer cells PC3 (Figure 1B). We used BPHL^CA ^and BPH^V ^cells for in vitro invasion assays with or without treatment with ilomastat (GM6001), a broad-spectrum hydroxamate inhibitor of MMP-2 and MMP-9 [35] and also MT1-MMP [36] (Figure 1). Our Invasion assay results indicated that BPHL^CA ^cells had a significantly higher percentage (4-5-fold) of invaded cells compared to control cells (BPH^V^) (Figure 1C). Upon treatment with MMP inhibitor GM6001 there was a significant decrease in invasion of BPHL^CA ^cells compared to vehicle treated cells (Figure 1D), which strongly suggests that MMPs are involved in LIMK1-induced cell invasion.

### LIMK1 expression was associated with increased secretion of pro-MMP-2 and pro-MMP-9 in the conditioned media

**Figure 2:**
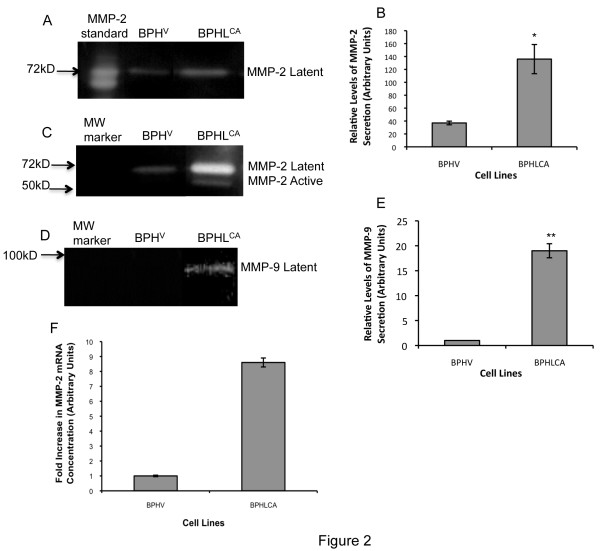
**Increased secretion of MMP-2 and MMP-9 in cells expressing LIMK1**. Gelatin zymography of secreted MMP-2 (A and C) and MMP-9 (D) in the conditioned media of BPHL^CA ^and BPH^V ^cells. A) Relative gelatinotytic activity of latent MMP-2. B) Densitometric analysis of relative gelatinolytic activity of MMP-2. C). Zymography showing latent, active or partially active MMP-2 in concentrated conditioned media of each cell line. Values are Mean ± SD of three experiments. * P = 0.010 (BPHL^CA ^vs. BPH^V ^cells). D): Gelatinolytic activity of pro-MMP-9 E): Densitometric analysis of the relative intensity of the gelatinoic bands of pro-MMP-9. Values are Mean ± SD of three experiments. ** P = 0.001 (BPHL^CA ^vs. BPH^V ^cells). F) Quantitative real-time PCR analysis of MMP-2 mRNA. Fold difference in mRNA concentrations in BPHL^CA ^compared to BPH^V ^cells. Data was calculated using normalized average ΔCT values for BPH^V ^and BPHL^CA ^cells. Values are Means ± SD of three independent analyses.

Because the hydroxamate inhibitor ilomastat inhibited LIMK1 induced invasion we intended to study the levels of secretory MMP-2 and MMP-9 in cells expressing LIMK1. Enhanced expression of MMPs has been correlated with increasing malignancy especially increased expression and activities of MMP-2 and MMP-9 are involved in the process of prostate cancer invasion and metastasis [25,37]. To examine this, we used BPHL^CA ^or BPH^V ^cells for analysis of MMP-2 and MMP-9 activities by zymography (Figure 2). In BPHL^CA ^cells, increased gelatinolytic activities of MMP-2 and MMP-9 were noted. Concentration of both latent and active forms for MMP-2 and the latent form of MMP-9 was increased in these cells (Figure 2A-E). In comparison, very low levels of latent and active forms of MMP-2 were noted in BPH^V ^cells (Figure 2A, B and 2C). To determine if LIMK1 expression is responsible for alteration in the expression of mRNAs of MMP-2 and MMP-9, we performed quantitative real-time RT-PCR analysis of the steady-state mRNA. Our results showed a 10-fold increase in MMP-2 mRNA concentration in BPHL^CA ^cells compared to BPH^V ^control cells (Figure 2F). We did not see any increase in the mRNA concentration of MMP-9 in these cells (data not shown).

### LIMK1 expression positively correlated with expression of MT1-MMP in prostate cancer cells

**Figure 3:**
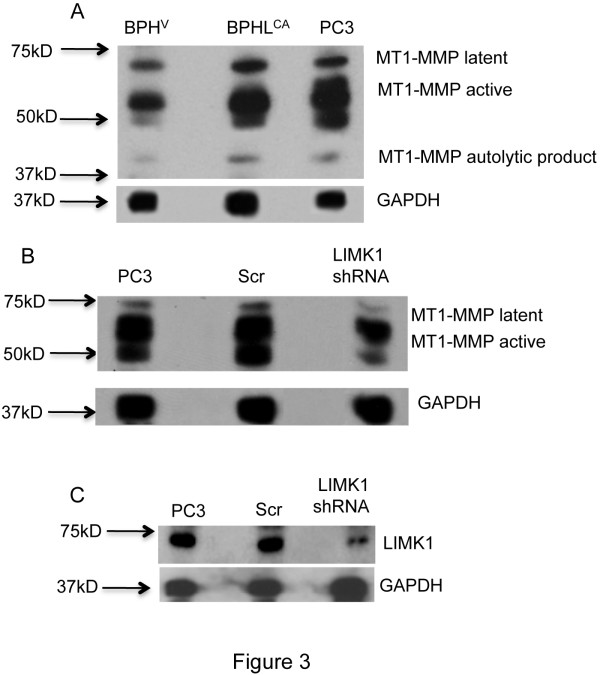
**Expression of LIMK1 associated with increased expression of MT1-MMP**. A) Expression of Pro-MT1-MMP (66 kD) and active MT1-MMP (57 kD) in BPH cells expressing LIMK1. Western blot analysis of MT1-MMP using anti-MT1-MMP antibodies in total protein extracts of BPH cells expressing LIMK1 phosphomimic mutant (pLIMK1). PC3 cell, that express high levels of LIMK1, also showed higher amounts of expression of latent and active MT1-MMP. B) Immunoblot analysis of MT1-MMP in these cells showing that inhibition of LIMK1 expression reduced MT1-MMP. C) Western blot analysis of LIMK1 using anti-LIMK1 antibodies in total extracts of wild type and transfected PC3 cells prepared at 72 hrs post transfection with LIMK1 shRNA or scrambled (scr) RNA expressing vector showing knock down of LIMK1 in PC3 cells. GAPDH was used as the loading control in all western blots.

Activation of MMP-2 is mediated by MT1-MMP, which is also a target of ilomastat. Therefore, next we studied the effect of expression of LIMK1 on MT1-MMP concentration in prostate cancer cells. In prostate cancers, MT1-MMP expression has been shown to correlate with the tumor stages and metastasis of prostate cancer cells in xenograft animal models [38]. MT1-MMP degrades several components of the ECM and this degradative activity is enhanced by activation of latent MMP-2. We used BPHL^CA^, BPHL^V ^and PC3 cells for analysis of MT1-MMP expression using MT1-MMP antibodies against the hinge region (ThermoFisher), which recognize all forms of MT1-MMP. Our immunoblot results showed increased expressions of the latent, active and 43 kD autolytic forms of MT1-MMP in BPHL^CA ^and PC3 cells compared to the parental BPH-1 cells (Figure 3A). Quantitative real-time RT-PCR analysis of the steady-state mRNA also showed an increase (~8-fold) in mRNA levels of MT1-MMP in BPHL^CA ^cells compared to BPH^V ^cells (data not shown). To confirm the involvement of LIMK1 in expression of MT1-MMP, we conducted LIMK1 shRNA-mediated down regulation of LIMK1 in PC3 cells. We used four different constructs for transfection to identify the one with maximum inhibition (shRNA 3) compared to nonspecific (scr) shRNA (Figure 3C and additional file 1). Short hairpin RNA induced down regulation of LIMK1 showed a significant reduction in both active and latent MT1-MMP concentrations in PC3 cells, which was not noted following expression of scr shRNA (Figure 3B). This observation indicates a positive correlation between LIMK1 and MT1-MMP expressions in these cells.

### LIMK1 and MT1-MMP are overexpressed in cancerous prostate

**Figure 4:**
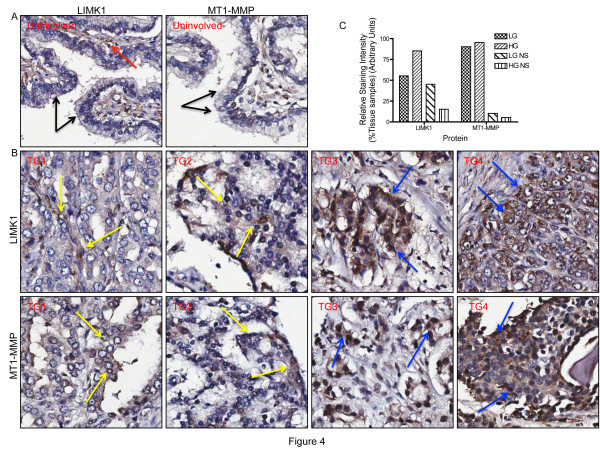
**Expression analysis of LIMK1 and MT1-MMP in clinical specimens**. A) Very low to no staining (black arrows) of LIMK1 and MT1-MMP in luminal cells and weak (red arrow) staining of LIMK1 in some of the basal cells in the normal prostate (Mag. × 200). B) Representative images of prostate tumors of different grades. Upper panels: Weak staining of LIMK1 (yellow arrows) in grade 1 (TG1) and grade 2 (TG2) tumors (left two boxes); Strong nuclear and cytoplasmic staining of LIMK1 in cancerous areas (blue arrows) of grade 3 (TG3) and grade 4 (TG4) tumors (right two boxes). Lower panels: Left two boxes: Weak staining of MT1-MMP (yellow arrows) in grade 1 (TG1) and grade 2 (TG2) tumors; Right two boxes: Strong nuclear and cytoplasmic staining of MT1-MMP in tumor areas could be seen (blue arrows) in grade 3 (TG3) and grade 4(TG4) tumors (Mag. × 200). C) Graphical representation of the relative staining intensity of LIMK1 and MT1-MMP in low-grade (LG) and high-grade (HG) tumors. LGNS and HGNS: Very low to no staining.

To understand the clinical relevance of LIMK1 and MT1-MMP overexpression, we examined the expression profiles of LIMK1 and MT1-MMP in clinical specimens using immunohistochemistry of formalin-fixed paraffin embedded prostate tumors tissues. We noted increased expression of LIMK1 and MT1-MMP in the tissues from the same tumors showing adenocarcinoma with Gleason scores 9/10 compared to the normal epithelium (Figure 4). All of the positive staining (weak to strong positive) of LIMK1 were located in cytoplasm or cytoplasm/nucleus in tumor cells of prostate adenocarcinoma (Figure 4B), while only weak staining (0-1+) in the cytoplasm of the glandular epithelium was noted in normal prostate tissues (Figure 4A). The intensity and the number of cytoplasm or nucleus stained positive cells as determined by IHC analysis software (data not shown) were in agreement with the pathologist's evaluation of scales of positive staining (0 to 3+) in most of cases. For MT1-MMP, positive staining (weak to strong positive) was present in the cytoplasm (majority) and in the nucleus (small numbers) in tumor cells of prostate adenocarcinoma (Figure 4B), whereas only weak staining (0-1+) in the cytoplasm or nuclei of the glandular epithelium could be seen in normal prostate tissues (Figure 4A). The intensity and the number of cytoplasm or nucleus stained positive cells as determined by IHC analysis software also were in agreement with the pathologist's analysis of scales of positive staining (0 to 3+) in most of the cases. Comparison of staining intensity with the tumor grades showed stronger staining of both antibodies in tumors with higher grades than that in tumors of lower grades (Figure 4C). A positive correlation in the nuclear expression (staining) of LIMK1 and MT1-MMP in grade 3 and grade 4 tumors, which increased from 20% (grade 3 tumors) to 40% (LIMK1) and 45%(MT1-MMP)(grade 4 tumors) of the tumor samples stained, was also noted (additional file 2). No correlation could be seen in the cytoplasmic expression of these proteins in these tumors. Although increased expression of MT1-MMP in prostate adenocarcinoma has already been reported our study showed overexpression of both LIMK1 and MT1-MMP in the same tumor samples, which supports our in vitro studies and shows an association between LIMK1 and MT1-MMP expressions in clinical samples.

### LIMK1 colocalizes and physically associates with MT1-MMP

**Figure 5:**
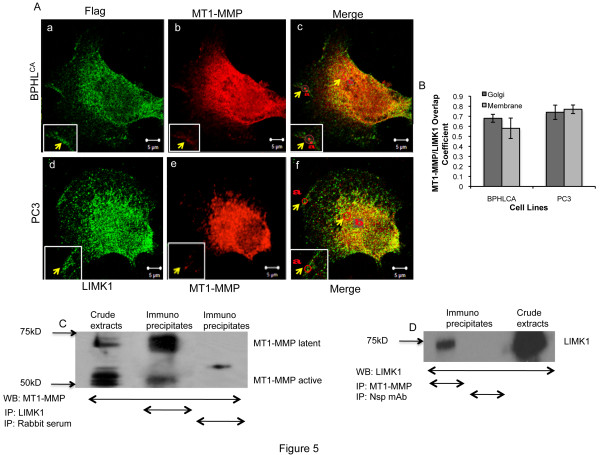
**Subcellular localization and physical association of LIMK1 with MT1-MMP**. A) Immunofluorescence analysis of LIMK^T508EE ^and MT1-MMP in BPHL^CA ^(a-c) and PC3 (d-f) cells. a-c: Co-localization of MT1-MMP (red) and LIMK1 (green)(merged image) in these cells was mainly to the perinuclear regions at the ER/Golgi area. Colocalization of MT1-MMP with LIMK1 was also at the plasma membranes (yellow arrows). These cells showed intense staining and accumulation of LIMK1 in the ruffling membranes (Insert: yellow arrows). Scale bar 5 μm. d-f: Colocalization of LIMK1 and MT1-MMP in PC3 cells. Immunofluorescence analysis of MT1-MMP and LIMK1 showed strong staining of both proteins in the Golgi areas and in transport vesicles (yellow arrows). Scale bar 5 μm. B) Analysis of colocalization using overlap coefficient of actual pixels in designated areas (red circles) at the Golgi region and at the membrane for both BPHL^CA ^and PC3 cells. C) Coimmunoprecipitation (Co-IP) and D: Reverse Co-IP of MT1-MMP and LIMK1 using anti-LIMK1 (mouse monoclonal) and anti-MT1-MMP (rabbit polyclonal) antibodies and PC3 cell extracts (500 μg) showing pull down of MT1-MMP and LIMK1 in immunoprecipitates. Nsp mAb: Nonspecific mouse monoclonal antibodies. Rabbit serum and nonspecific mouse mAb were used as the negative controls.

Physical interaction between LIMK1 and MT1-MMP was studied next using immunofluorescence and immunoprecipitation experiments. Intracellular localization of MT1-MMP and LIMK^T508EE ^in BPHL^CA ^and PC3 cells was monitored using anti-MT1-MMP (Neomarker) and anti-Flag or anti-LIMK1 antibodies. Our results showed colocalization of MT1-MMP and LIMK^T508EE ^(BPHL^CA^) or LIMK1 to the perinuclear region and in the plasma membrane of both cell types (Figure 5A). These cells also showed intense staining in the Golgi areas and accumulation of LIMK1 in the ruffling membranes (insert, BPHL^CA ^and PC3). Quantitative analysis of overlapping pixels and intensity using Zeiss Zen 2009 software confirmed colocalization of MT1-MMP and LIMK1 in these cells (Figure 5B and additional file 3). A physical association between LIMK1 and MT1-MMP was also noted in coimmunoprecipitation and reverse coimmunoprecipitation experiments using PC3 cell extracts, which showed that LIMK1 interacted either directly or indirectly, with both active and latent MT1-MMP (Figure 5C and 5D).

### LIMK1 facilitates transport of MT1-MMP to the plasma membrane through Golgi vesicles

**Figure 6:**
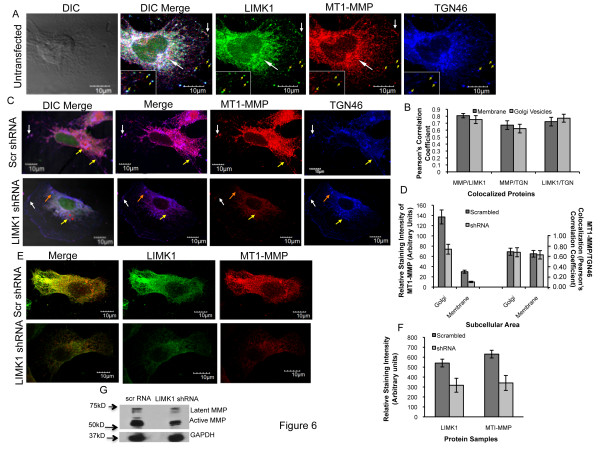
**LIMK1 regulates vesicular transport of MT1-MMP**. A) Immunolocalization of MT1-MMP and LIMK1 showed strong staining of both proteins in the Golgi areas (White staining in the DIC merge and long white arrows) along with Golgi marker: TGN46. Yellow arrows: Colocalization of MT1-MMP and LIMK1 in Golgi vesicles at various distance towards plasma membrane: White arrows: Colocalization of MT1-MMP and LIMK1 to the plasma membrane. B) Analysis of Pearson's correlation coefficient of colocalization between LIMK1 and TGN46, MT1-MMP and TGN46, and LIMK1 and MT1-MMP in Golgi vesicles and in the membrane areas (insert in A: yellow arrows), C) Loss of expression and plasma membrane localization of MT1-MMP in PC3 cells following knock down of LIMK1 expression. Immunofluorescence analysis of MT1-MMP, and TGN46 expression and localization in PC3 cells transfected with scrambled RNA (green) (upper panel) or LIMK1 shRNA (green)(lower panel) expressing vectors. Upper panel: Yellow arrows: Colocalization of MT1-MMP with TGN46 in Golgi vesicles moving towards the plasma membrane, White arrow: Targeting of MT1-MMP to the plasma membrane. Lower panel: Colocalization of MT1-MMP and TGN46 to the perinuclear region (yellow arrow) but not in the transport vesicles (orange arrow). White arrow: Transport of TGN46 positive Golgi vesicles to the plasma membrane. No targeting of MT1-MMP could be noted in these cells. A significant reduction in the MT1-MMP concentration was also evident in these cells. Scale: 10 μm. D) Quantitative analysis of the staining intensity of MT1-MMP in the Golgi vesicles (red circles in DIC merge: yellow arrow) and in the membrane (entire membrane) in scrambled RNA and LIMK1 shRNA transfected PC3 cells. Pearson's correlation coefficient analysis confirms colocalization between MT1-MMP and TGN46 in Golgi vesicles at the same region used for the analysis of staining intensity. E and F) Immunofluorescence and quantitative analyses of the reduction of LIMK1 expression in LIMK1 shRNA but not scrambled RNA expression vector transfected cells. G) Western blots of MT1-MMP in the lysates of PC3 cells expressing scrambled shRNA or LIMK1 shRNA used for immunolocalization assays.

Our imunofluorescence images showed possible localization of LIMK1 and MT1-MMP in the Golgi vesicles. To confirm that LIMK1 colocalizes with MT1-MMP to the Golgi vesicles, we used antibodies against Trans-Golgi Network Golgi marker TGN46. Triple label immunofluorescence analysis showed colocalization of LIMK1, MT1-MMP and TGN46 to the Golgi transport vesicles in the perinuclear region and between TGN and the plasma membrane (Figure 6A). Pearson's correlation coefficient analysis of colocalization between MT1-MMP and TGN46, MT1-MMP and LIMK1 and LIMK1 and TGN46 at the Golgi vesicles and in the membrane area confirms vesicular transport of these proteins (Figure 6B and additional file 4A and 4B). Down regulation of LIMK1 by shRNA in PC3 cells showed a substantial reduction in MT1-MMP staining and its restricted localization mainly to the perinuclear regions compared to cells expressing nonspecific shRNA (Figure 6C). Quantitative analysis of staining intensity in the Golgi and in the membrane confirmed reduction of MT1-MMP in the Golgi vesicles as evident from correlation coefficient analysis between TGN46 and MT1-MMP in these areas (Figure 6D and additional file 4B). Immunofluorescence and quantitative intensity analysis confirmed down regulation of MT1-MMP and LIMK1 but not TGN46 in LIMK1 shRNA transfected cells (Figure 6E and 6F and additional file 4C). No reduction in MT1-MMP levels was noted in cells transfected with scrambled RNA. Immunoblot analysis of shRNA transfected cells confirmed down regulation of both latent and active MT1-MMPs in these cells (Figure 6G). Colocalization of MT1-MMP with TGN46 was noted in the perinuclear regions but not in the Golgi vesicles moving towards the plasma membrane. This observation indicates an inhibitory effect of LIMK1 knockdown on the vesicular transport of MT1-MMP to the plasma membrane.

### Expression of LIMK1 increased surface localization of MT1-MMP

**Figure 7:**
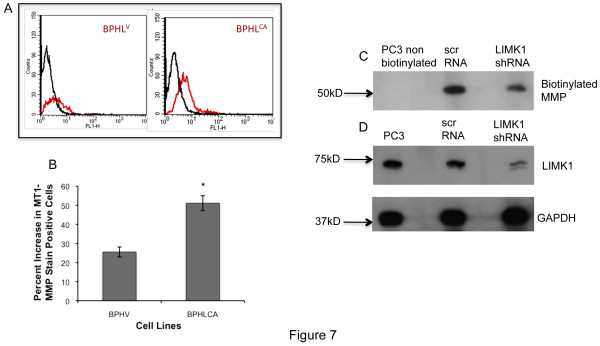
**LIMK1 expression positively regulates surface localization of MT1-MMP in BPH and PC3 cells**. A) Flow cytometric analysis of MT1-MMP cell surface expression in transfected BPH cell lines. Two-parameter histogram of the surface staining of MT1-MMP with fluorescence intensities in the X-axis and number of cells in the Y-axis in BPH cells expressing LIMK1. Black histogram represents unstained cells. Red histogram represents population of fluorescent cells within each sample. B) Quantitative analysis of fluorescent cells. For densitometric analysis, the BPHL^CA ^cells that were emitting fluorescence were gated out from the nonfluorescent cells and calculated as the percentage of the fluorescent control (BPH^V^) cells. Data represents the mean ± SD of three independent experiments. *P = 0.0005. C) Surface biotinylation of MT1-MMP following knock down of LIMK1 in PC3 cells. Immunoblot of biotinylated MT1-MMP in the streptavidin bead bound MT1-MMP. Lane 1 non-biotinylated cells, Lane 2: control shRNA expressing cells, Lane 3: LIMK1 shRNA expressing cells. D) Immunoblot analysis of LIMK1 in PC3 cells expressing control shRNA or LIMK1 shRNA used for surface biotinylation assays. Data show representative images of three independent experiments.

To determine any change in the activation status of MT1-MMP upon expression of LIMK1, we monitored surface localization of MT1-MMP in these cells. Activation of MT1-MMP requires cleavage of the propeptide by furin like convertases, the active form of MT1-MMP, and its insertion into the plasma membrane, therefore the plasma membrane associated form is considered to be the active form of MT1-MMP. To confirm increased activation of MT1-MMP, we studied surface localization of MT1-MMP in cells expressing LIMK1 using flow cytometry and surface biotinylation assays (Figure 7). We used antibodies against MT1-MMP, which recognize the catalytic domain of MT1-MMP that is exposed to the extracellular side of the plasma membrane and could be detected in intact cells. Our results indicated that the BPHL^CA ^cells expressed a higher amount of MT1-MMP on the surface (Figure 7A) as shown by the right shift of the red histogram compared to BPH^V ^cells. Densitometric analysis of the number of fluorescent cells showed a two-fold increase in MT1-MMP expression in BPHL^CA ^cells compared to BPH^V ^cells (Figure 7B). Surface localization of MT1-MMP and its relation with expression of LIMK1 was further studied in PC3 cells by surface biotinylation followed by western blotting using MT1-MMP antibodies against the hinge region (Millipore). Our result showed expression of biotinylated MT1-MMP at the surface of PC3 cells decreased following knockdown of LIMK1 compared to control shRNA expressing cells, and confirmed the role of LIMK1 in regulation of surface localization of MT1-MMP (Figure 7C and 7D).

### Expression of LIMK1 was associated with increased transcriptional activation of MT1-MMP

**Figure 8:**
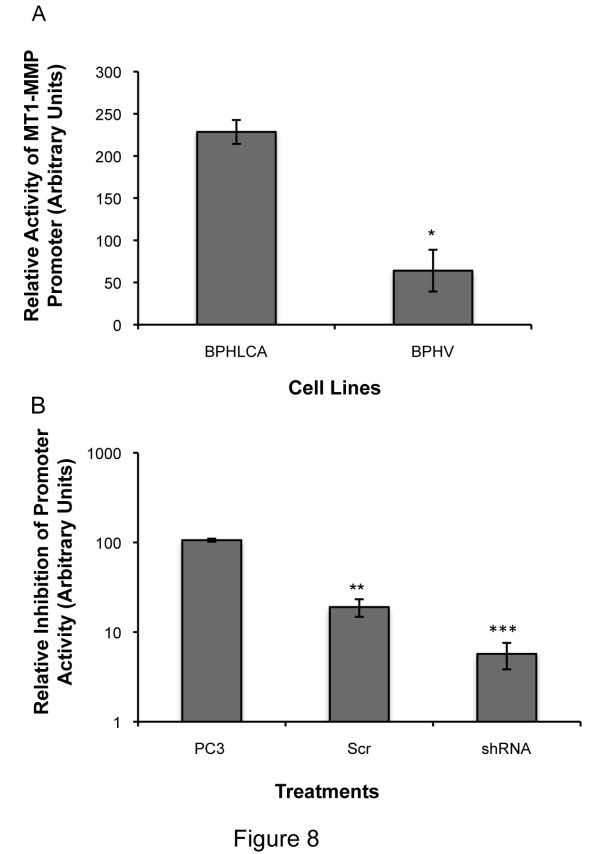
**Effect of LIMK1 on transcription activation of MT1-MMP**. A) Relative luciferase activity in BPHL^CA ^and BPH^V ^cells transfected with MT1-MMP promoter luciferase constructs. B) Relative luciferase activity in PC3 cells transfected with MT1-MMP promoter luciferase construct alone or in combination with scrambled shRNA or LIMK1 shRNA expressing plasmids. Results show Mean ± SD of at least three separate experiments. *P = 0.0075 (BPHL^CA ^vs. BPH^V^), **P = 0.004 (PC3 scr shRNA vs. PC3LIMK1 shRNA), ***P = 0.0005 (PC3 vs. PC3LIMK1 shRNA).

MT1-MMP and other MMPs are overexpressed in advanced prostate cancers and also in invasive PC3 cells [39]. Specifically, MT1-MMP expression is regulated in prostate cancer cells based on the aggressiveness of the cell type [40]. Increased expression of MT1-MMP in PC3 cells is mediated by transcriptional activation of MT1-MMP promoter through Sp1 transcription factor activated by the components of AKT/JNK pathway. PI3K/AKT is also capable of activating LIMK1 through direct activation of PAK1/PAK4 [41] in response to treatment with bone morphogenic protein II (BMPII). In this study, we examined the effect of down regulation of LIMK1 on MT1-MMP promoter activation using luciferase reporter assays. We used transfected BPHL^CA ^and BPH^V ^cells, and PC3 cells for transient transfection of luciferase expression plasmids driven by the full-length (7.2 kb) MT1-MMP promoter (Figure 8). Dual luciferase assays showed a 3-4-fold increase in luciferase expression in BPHL^CA ^cells compared to BPH^V ^cells, suggesting a positive correlation between expression of LIMK1 and transcriptional activation of MT1-MMP (Figure 8A). This assumption was further supported by the results obtained following knock down of LIMK1. PC3 cells transfected with shRNA constructs of LIMK1 showed a significant reduction (95%) in luciferase expression compared to untransfected PC3 cells (Figure 8B). Although some off target effects of scr shRNA were observed the reduction of luciferase expression between untransfected and LIMK1 shRNA transfected cells was much higher and consistently observed in experimental repeats. Other LIMK1 shRNA constructs, which were not effective in reduction of LIMK1, showed similar or higher activation of MT1-MMP promoter as noted in scrambled RNA transfected cells. This rules out the possibility of the off target effect of LIMK1 shRNA 3 (used for all experiments) (additional file 5). This observation indicates that expression of LIMK1 has a stimulatory effect on transcription of MT1-MMP.

## Discussion

LIMK1 being an actin and microtubule modulatory protein is likely to be involved in acquisition of an invasive phenotype commonly noted in tumors exhibiting advanced stages of malignancy. Accordingly, earlier studies including ours clearly demonstrated an important role of LIMK1 in induction of invasion and metastasis of prostate and breast cancer cells and tumors [2-4]. More recently, role of LIMK1 in induction of metastasis in pancreatic cancer in zebrafish xenograft assays [42]; and mesenchymal and ameboid modes of invasion of fibrosarcoma cells in 3D matrices [43,44] were shown. These studies further strengthened the importance of LIMK1 and Rho/Rock signaling pathway in generation of protrusive forces of tumor cells through collagen matrices.

Our studies presented here demonstrated that LIMK1 is involved in regulating MT1-MMP functions at various levels. The role of MT1-MMP in invasion and metastasis through direct and indirect collagenolytic activities is well documented therefore it is likely that the critical role of LIMK1 in facilitation of cell motility and invasion is at least partly mediated through modulation of MMP functions. We confirmed the association of MMPs such as MT1-MMP, MMP-2 and MMP-9 in LIMK1-induced invasion of BPHL^CA ^cells in invasion assays. Expression of phosphomimic LIMK1 in BPH-1 cells changed their noninvasive phenotype to invasive ones, but use of the hydoxamate inhibitor (GM6001) of MMPs, specifically MT1-MMP, MMP-2 and MMP-9 completely abrogated the invasive property of these cells. To our knowledge, this is the first report showing a functional link between LIMK1 and MT1-MMP.

The question that we asked next is that if LIMK1 expression also increased the proteolytic function of MMPs. Indeed, our results showed that processing of pro-MMP-2 to its active form was increased in BPHL^CA ^cells (Figure 2). In addition, there was an overall increase in secreted MMP-2 (latent) as commonly noted in invasive prostate cancer cells. We investigated whether LIMK1 expressing cells also have a higher expression of mRNAs of pro-MMP-2, which was also the case and a significant increase in MMP-2 mRNA was noted in these cells. Increased expression of LIMK1, either ectopically or endogenously, was associated with a significant increase in expressions of the latent and active forms of MT1-MMPs, which were substantially diminished upon knockdown of LIMK1. It is speculated that increased expression of the latent MT1-MMP resulted in increased levels of active MT1-MMP as seen in BPHL^CA ^and PC3 cells. The correlative expression profile of LIMK1 and MT1-MMP was also detected in clinical samples pathological reports of which showed higher tumor grades. In addition, most of tumor cells of prostate adenocarcinoma had higher cytoplasmic and nuclear expression of LIMK1 and MT1-MMP, compared to normal prostate tissues. A number of studies showed increased expression of MT1-MMP in advanced cancers including prostate cancer. Increased expression of LIMK1 in luminal cells has been reported also in advanced prostate tumors. Our study shows increased expression of both proteins in the same tumor samples, which suggests a clinical relevance of overexpression of both LIMK1 and MT1-MMP.

Our studies further provide evidence that LIMK1 physically interacts with MT1-MMP and regulates its vesicular transport to the plasma membrane. Colocalization of MT1-MMP and LIMK1 was in abundance in the Golgi areas at the perinuclear region in both PC3 and BPHL^CA ^cells. In addition, distinct vesicles at various distances between perinuclear region to the plasma membrane with colocalized LIMK1, MT1-MMP and TGN46 were seen in PC3 cells. This observation indicating a role of LIMK1 in vesicular trafficking is in support of earlier reports showing that LIMK1 regulates endocytic or exocytic vesicular transport in endothelial cells for transport of SREBP cleavage activating protein (SCAP) [45]. Earlier studies also showed that LIMK1 modulates Golgi dynamics through protein-protein interaction through its LIM domain and trafficking of Golgi transport vesicles between ER and Golgi in primary neuronal cells [46]. Recent studies by Nishimura et al [47] indicated a role of LIMK1 in regulating endocytic trafficking of EGFR wherein LIMK1 delays internalization of EGFR bound EGF, thereby maintaining sustained activation of EGF/EGFR axis in invasive tumor cells. Nonetheless, it is not clear if LIMK1 also regulates internalization of MT1-MMP and requires further study. Importantly, inhibition of LIMK1 dramatically reduced plasma membrane targeting of MT1-MMP (Figure 6), which confirms a distinct regulatory role of LIMK1 in vesicular transport of MT1-MMP for its surface localization.

Surface localization of MT1-MMP is another essential event for MT1-MMP to be functionally active for its collagenolytic activities either directly or through increased activation of soluble pro-MMP-2 through the ternary complex formation (MT1-MMP/TIMP-2/pro-MMP-2), and pro-MMP-9 through activation of MMP-2/TIMP2 axis [14,23]. In support of our immunoblot results, increased surface localization of MT1-MMP was noted in BPHL^CA ^cells expressing LIMK^T508EE ^as shown by flow cytometry. Surface biotinylation assays in PC3 cells following knockdown of LIMK1 further confirmed the role of LIMK1 in MT1-MMP surface localization. We speculate that LIMK1 regulates surface localization of MT1-MMP through its physical interaction with MT1-MMP, as our immunofluorescence analysis and coimmunoprecipitation studies confirmed such interaction. It is possible that interaction between LIMK1 and latent MT1-MMP helps the proteolytic processing of MT1-MMP and its targeting to the plasma membrane.

Our studies using luciferase reporter assays showed that LIMK1 expression increased activation of MT1-MMP promoter in BPHL^CA ^cells and knockdown of LIMK1 significantly reduced luciferase expression in PC3 cells. This result indicates that LIMK1 has a regulatory role in transcription of MT1-MMP and thereby increases pro-MT1-MMP levels when overexpressed in cells. Role of LIMK1 in promoter activation has been reported earlier, which showed that LIMK1 expression increased activation of uPA promoter in breast cancer cells [4]. However, how LIMK1 induces transcriptional activation of MT1-MMP is not clear and studies are underway to determine the mechanism of LIMK1-induced increased transcription of MT1-MMP. To this end, the importance of this study lies in the realm of a possibly better therapeutic approach for metastatic cancer by inhibition of LIMK1 instead of MMP inhibitors which showed higher toxicity.

## Competing interests

The authors declare that they have no competing interests.

## Authors' contributions

TT carried out the studies involving invasion assays, zymography, RT-PCR and flow cytometry. She also generated stable cell lines expressing LIMK1 constructs. RO performed all microscopy, quantitative analysis, LIMK1 knock down experiments, surface labeling experiments and reporter assays. Both TT and RO were involved in data analysis, participated in manuscript writing and contributed equally in this study. RC conceived the idea of the study and involved in conceptual study design, writing manuscript, surface-labeling experiment with RO, trouble shooting and data analysis. All authors read and approved the final manuscript.

## Supplementary Material

Additional file 1 **Western blot analysis of knock down of LIMK1 using shRNA constructs**. Western blots of LIMK1 using anti-LIMK1 antibodies in total extracts of wild type and transfected PC3 cells prepared at 72 hrs post transfection with different constructs of LIMK1 shRNA or scrambled (scr) RNA expressing vector showing knock down of LIMK1 in PC3 cells. GAPDH was used as the loading control. Click here for file

Additional file 2 **Quantitative analysis of nuclear and cytoplasminc staining of LIMK1 and MT1-MMP**. Analysis of expression patterns of LIMK1 and MT1-MMP in prostate tumor tissues. A) Nuclear staining in grade 3 and grade 4 tumors. B) Cytoplasmic staining in grade 3 and grade 4 tumors. Data shows a distinct increase in percent of tumors with higher pathological grade exhibiting nuclear staining of both proteins. Click here for file

Additional file 3 **Colocalization of LIMK1 and MT1-MMP in the Golgi and plasma membrane areas**. Scatter plots of colocalization of LIMK1 and MT1-MMP in the selected areas in the membrane and in the Golgi region in BPHL^CA ^and PC3 cells. Presence of pixels in the quadrant 3 indicates colocalization. Click here for file

Additional file 4 **Quantitative analysis of colocalization of TGN46, LIMK1 and MT1-MMP in the Golgi vesicles**. Quantitative analysis of colocalization of LIMK1, MT1-MMP and Golgi marker TGN46 in the Golgi vesicles and in the membrane. A). Scatter plots of colocalization of LIMK1/TGN46, MT1-MMP/TGN46 and LIMK1/MMP in the same areas (yellow arrows in Figure 5A). Presence of pixels in upper right quadrant indicates colocalization of two proteins. B) Analysis of overlap coefficient between LIMK1 and MT1-MMP following knockdown of LIMK1. Data represent average correlation coefficient ± SD of 5-6 different subcellular areas selected in PC3 cells transfected with LIMK1 shRNA or scrambled RNA. Data shows reduced overlaps between LIMK1 and MT1-MMP in LIMK1 shRNA expressing cells. C) Relative staining intensity of TGN46 in the Golgi and in the cell membrane with or without knock down of LIMK1. Data represent average correlation coefficient ± SD of 6 different subcellular areas chosen for the analysis. Data show no effects of knock down of LIMK1 on the staining intensity of TGN46. Click here for file

Additional file 5 **Analysis of MT1-MMP promoter activity following knock down of LIMK1**. Relative luciferase activity in PC3 cells transfected with MT1-MMP promoter luciferase construct alone or in combination with scrambled shRNA or four different LIMK1 shRNA expressing plasmids as shown in additional file 1. Results show Mean ± SD of at least three separate experiments. Click here for file
