# Supplementary figures and images for: LIM kinase1 modulates function of membrane type matrix metalloproteinase 1: implication in invasion of prostate cancer cells
Source: Mol Cancer. 2011 Jan 10;10:6. doi: 10.1186/1476-4598-10-6 (PMC3027192; doi:10.1186/1476-4598-10-6)

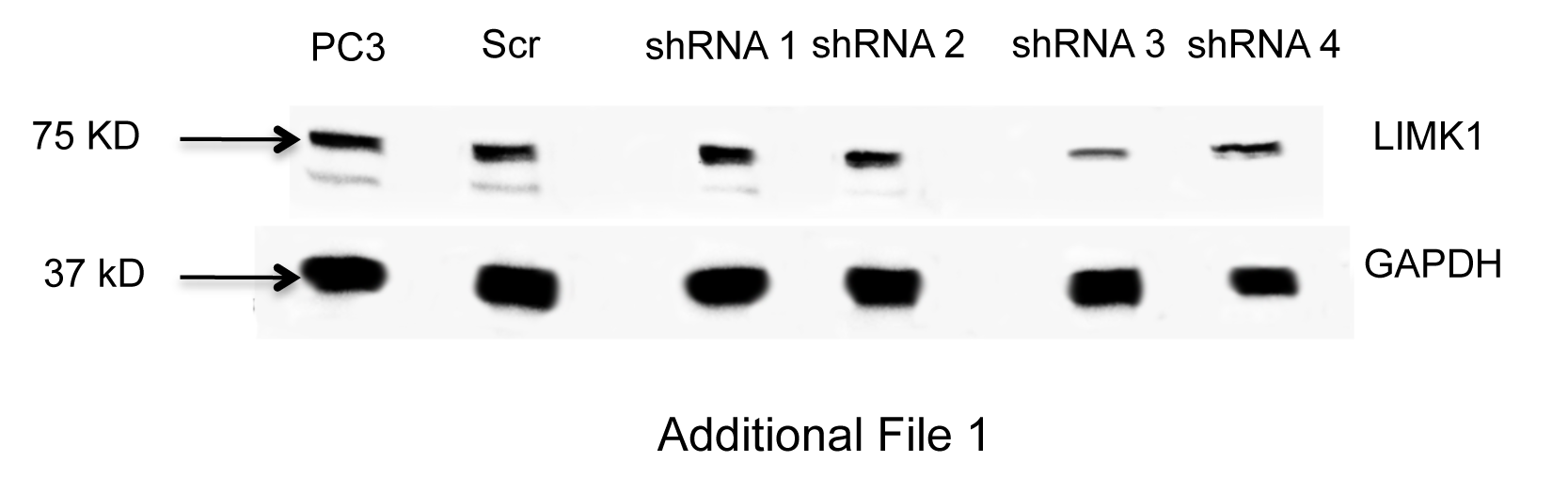

Supplement: Additional file 1 — Western blot analysis of knock down of LIMK1 using shRNA constructs. Western blots of LIMK1 using anti-LIMK1 antibodies in total extracts of wild type and transfected PC3 cells prepared at 72 hrs post transfection with different constructs of LIMK1 shRNA or scrambled (scr) RNA expressing vector showing knock down of LIMK1 in PC3 cells. GAPDH was used as the loading control. [file 1476-4598-10-6-S1.TIFF]

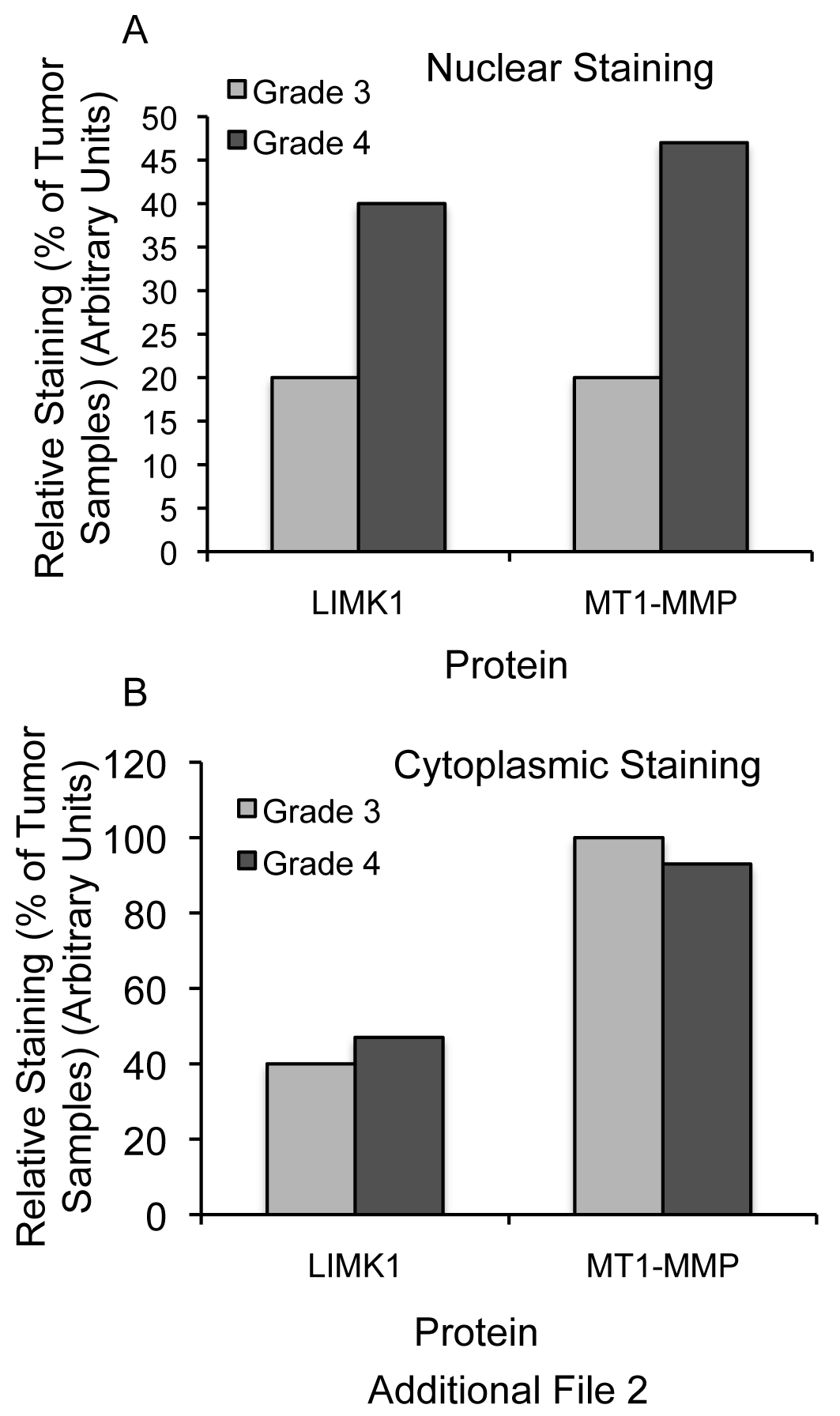

Supplement: Additional file 2 — Quantitative analysis of nuclear and cytoplasminc staining of LIMK1 and MT1-MMP. Analysis of expression patterns of LIMK1 and MT1-MMP in prostate tumor tissues. A) Nuclear staining in grade 3 and grade 4 tumors. B) Cytoplasmic staining in grade 3 and grade 4 tumors. Data shows a distinct increase in percent of tumors with higher pathological grade exhibiting nuclear staining of both proteins. [file 1476-4598-10-6-S2.TIFF]

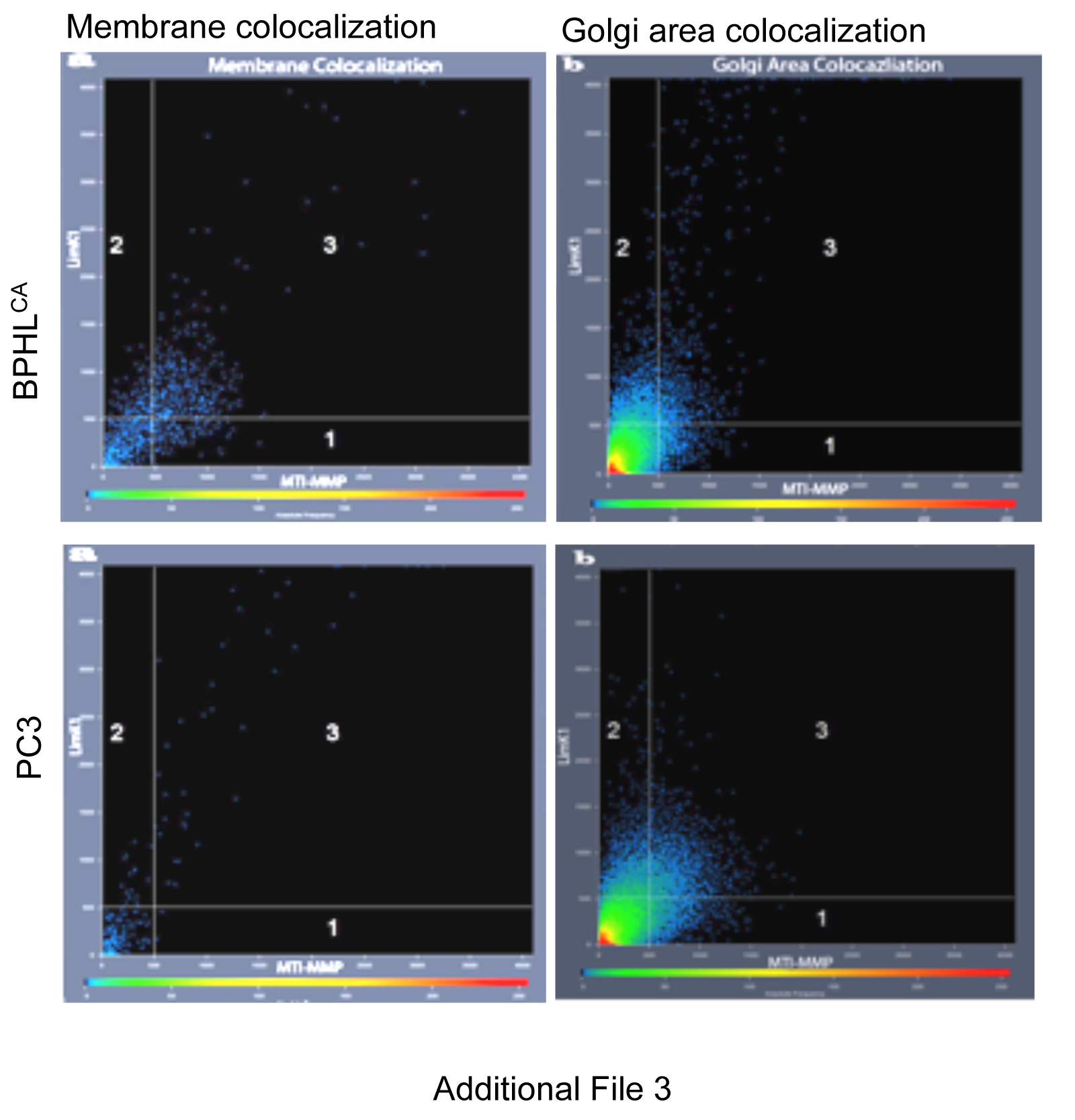

Supplement: Additional file 3 — Colocalization of LIMK1 and MT1-MMP in the Golgi and plasma membrane areas. Scatter plots of colocalization of LIMK1 and MT1-MMP in the selected areas in the membrane and in the Golgi region in BPHLCA and PC3 cells. Presence of pixels in the quadrant 3 indicates colocalization. [file 1476-4598-10-6-S3.TIFF]

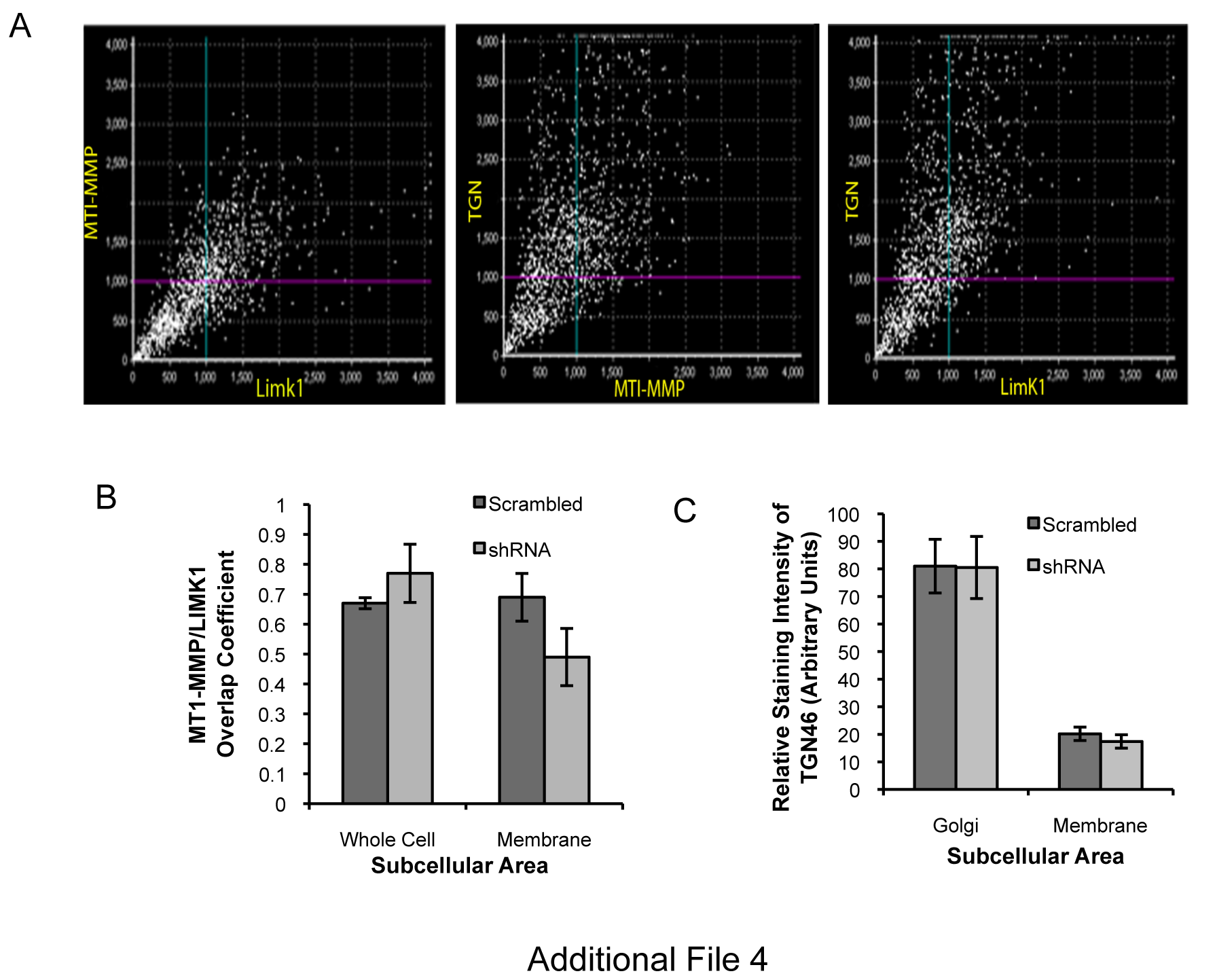

Supplement: Additional file 4 — Quantitative analysis of colocalization of TGN46, LIMK1 and MT1-MMP in the Golgi vesicles. Quantitative analysis of colocalization of LIMK1, MT1-MMP and Golgi marker TGN46 in the Golgi vesicles and in the membrane. A). Scatter plots of colocalization of LIMK1/TGN46, MT1-MMP/TGN46 and LIMK1/MMP in the same areas (yellow arrows in Figure 5A). Presence of pixels in upper right quadrant indicates colocalization of two proteins. B) Analysis of overlap coefficient between LIMK1 and MT1-MMP following knockdown of LIMK1. Data represent average correlation coefficient ± SD of 5-6 different subcellular areas selected in PC3 cells transfected with LIMK1 shRNA or scrambled RNA. Data shows reduced overlaps between LIMK1 and MT1-MMP in LIMK1 shRNA expressing cells. C) Relative staining intensity of TGN46 in the Golgi and in the cell membrane with or without knock down of LIMK1. Data represent average correlation coefficient ± SD of 6 different subcellular areas chosen for the analysis. Data show no effects of knock down of LIMK1 on the staining intensity of TGN46. [file 1476-4598-10-6-S4.TIFF]

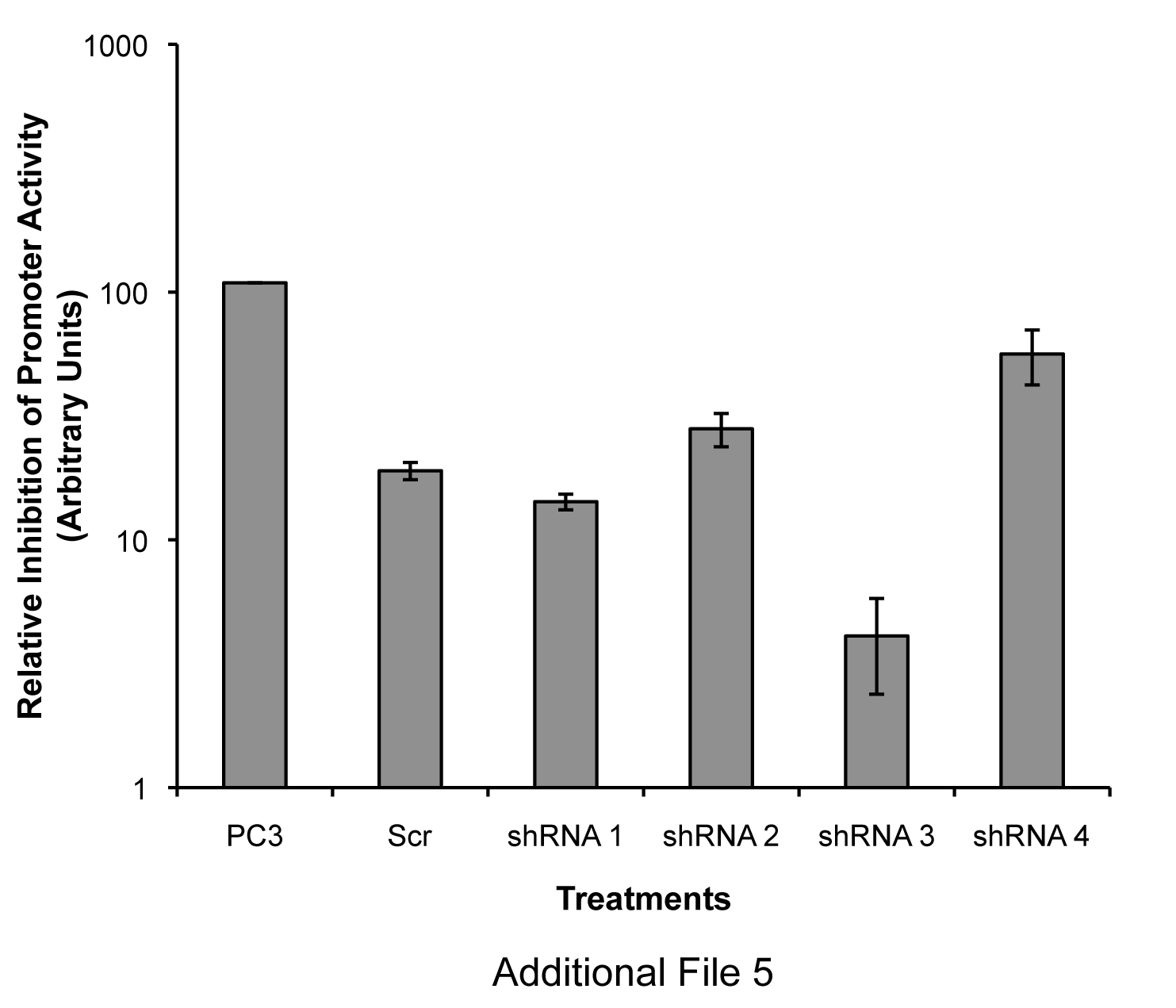

Supplement: Additional file 5 — Analysis of MT1-MMP promoter activity following knock down of LIMK1. Relative luciferase activity in PC3 cells transfected with MT1-MMP promoter luciferase construct alone or in combination with scrambled shRNA or four different LIMK1 shRNA expressing plasmids as shown in additional file 1. Results show Mean ± SD of at least three separate experiments. [file 1476-4598-10-6-S5.TIFF]
